# Early versus delayed postoperative extubation after elective neurosurgical treatment of brain metastasis

**DOI:** 10.1007/s00432-025-06278-8

**Published:** 2025-08-04

**Authors:** Logman Khalafov, T. Lampmann, M. Hamed, J. Dittmer, I. Maiseyeu, H. Alenezi, M. Jaber, H. Asoglu, M. Thudium, F. Lehmann, S. Ehrentraut, J. Poth, H. Vatter, M. Schneider, M. Banat

**Affiliations:** 1 Department of Neurosurgery, University Hospital Bonn, Venusberg Campus 1, Building 81, 53127 Bonn, Germany; 2 Department of Anesthesiology, University Hospital Bonn, Bonn, Germany

**Keywords:** Early extubation, Delayed extubation, Brain metastasis, Intensive care unit

## Abstract

**Introduction:**

It is generally assumed that early extubation after elective neurosurgical treatment of brain metastases (BMs) is associated with a lower rate of adverse events (AE), such as an increased rate of respiratory infections. The aim of this study is to investigate to what extent this association holds for the patient cohorts of our clinic who underwent elective intracranial surgery and whether in our experience early extubation (EE) was inferior to delayed extubation (DE).

**Material and methods:**

Between 2018 and 2020, 190 patients were surgically treated for BM in the authors’ neurosurgery department. Early extubation was defined as extubation immediately after surgery in the recovery room. The DE group was electively extubated after surgery in the intensive care unit. We analyzed demographic data, ASA status, blood loss, comorbidities, duration of surgery, blood transfusion, length of hospital stay, surgical-related complications and adverse events.

**Results:**

A total of 65 patients (34.2%) were extubated early. In the remaining 65.8% of patients extubation was delayed. In the univariate analysis, no statistical significance was found between the two groups, particularly with regard to complications. The only relevant difference was in the DE group, who had greater transfusion requirements (*p* = 0.037). The DE group showed more AE, but this was not significant in the multivariate analysis.

**Conclusions:**

Our data demonstrate that early extubation was justifiable and safe for our patients. Early extubation in the recovery room did not pose a risk of re-intubation immediately after elective neurosurgical resection of a brain metastasis.

## Introduction

Up to 15%-40% of patients with systemic tumor disease develop both cranial and spinal metastases in the course of their disease; this metastasis is an expression of the advanced stage of their tumor disease (Ernani and Stinchcombe 2019; Sung et al. 2021; Jacobs and Perrin 2001; Jenis et al. 1999; Brande et al. 2022; Barnholtz-Sloan et al. 2004; Alexandru et al. 2012). Some tumors metastasize more frequently to the central nervous system (brain metastasis [BM] and spinal metastasis) than others; these tumors include breast, prostate and bronchioloalveolar carcinomas (Berghoff et al. 2016; Page et al. 2020; Cagney et al. 2017; Zhang et al. 2020).

Surgical treatment of such BMs is now an established therapeutic procedure. The surgical response has several objectives: firstly, histological confirmation; secondly, removal of the space occupied by the metastasis and its surrounding brain oedema; and thirdly, prevention of other relevant complications of hydrocephalus or cerebral herniation (Vecht et al. 1993; Patchell et al. 1990; Boire et al. 2020). Systemic chemotherapy, immunotherapy and cerebral radiotherapy are used in addition to surgical interventions to improve overall survival (Achrol et al. 2019; Patchell 2003).

The question arises again and again as to where the patients should be monitored after surgical treatment: is early extubation and monitoring of the patients for a few hours in the recovery room and later transfer to intermediate care sufficient, or should the patients be monitored under controlled conditions in the intensive care unit (ICU)? Our internal clinic guideline gives clear recommendations for patients after epilepsy surgery (Bahna et al. 2022). However, there is a lack of data in the literature on the management of BMs after elective craniotomies and elective resections (Neumann et al. 2023). Some studies focus on the question of whether patients should be monitored postoperatively in the ICU or not (Gaudet et al. 2024; Cai et al. 2013). Patients with brain metastases are vulnerable and sometimes present at an advanced stage of the tumor, so careful perioperative planning is very important. This planning includes postoperative management with extubation after neurosurgical treatment of the brain metastases. Extubation—in the recovery room or in the intensive care unit—should simplify procedures and be safe and effective.

The aim of this study was to investigate whether delayed extubation (DE) was associated with a higher rate of postoperative adverse events in patients who had undergone elective intracranial surgery for BMs at our institution, and to assess whether early extubation (EE) was a feasible and safe alternative.

## Material and methods

### Patients and inclusion criteria

We conducted a retrospective monocentric data analysis at our neuro-oncological-neurosurgical center. This study is based on consecutive patients aged > 18 years who received primary surgical treatment for BM between 2018 and 2020 in the neurosurgery department at the University Hospital Bonn. Recommendations for post-surgery management were established through interdisciplinary consensus, occasionally coordinated with the treatment plans of the referring physicians (Schafer et al. 2021). The postoperative management protocol for brain metastases was changed at our center in 2019. We aimed to extubate all patients early and then monitor them on the ICU or IMC ward if possible.

A total of 190 patients were surgically treated for BM in the neurosurgery department. Patients were categorized into two distinct groups for further analysis: patients in the first group (EE) were extubated immediately after surgery in the recovery room. The second group (DE) was extubated after surgery in the ICU. We have been extubating patients in the recovery room since 2019. To enable us to make a comparison, patients from the previous year who were routinely extubated in the intensive care unit as planned were included in the analysis. After extubation, the patients were transferred to the intensive care unit for further monitoring.

Comprehensive clinical and neurological data were gathered, including the following parameters: age, sex, primary tumor type, BM location, details of the neurosurgical treatment, American Society of Anesthesiologists (ASA) score, clinical neurological scores, and functional status measured using the Karnofsky Performance Scale (KPS) upon admission, categorizing patients into KPS ≥ 70% or KPS < 70%, as previously described (Schuss et al. 2021; Hamed et al. 2023; Schweppe et al. 2023; Ilic et al. 2021). The Charlson Comorbidity Index (CCI) was employed to quantify patients’ comorbidity burden before undergoing surgery (Hamed et al. 2022; Schneider et al. 2020a; Lehmann et al. 2023).

Early in-house postoperative complications were assessed using a publicly available list of adverse events introduced by the Agency for Healthcare Research and Quality and the Center for Medicare and Medicaid Services and referred to as patient safety indicators (PSIs) and hospital-acquired conditions (HACs) (Al-Tehewy et al. 2020; Stocking et al. 2020; Horn et al. 2020; Schneider et al. 2020b). The list of possible PSIs includes iatrogenic pneumothorax, pressure ulcers, acute myocardial infarction, peri- and postoperative hemorrhage, pulmonary embolism, transfusion reactions, deep vein thrombosis, acute postoperative respiratory failure, postoperative sepsis, and wound dehiscence. HAC screening was performed for pneumonia, crushing injury, surgical site infection, manifestation of poor glycemic control (diabetic ketoacidosis, non-ketonic hyperosmolar coma, hyperglycemic coma), blood incompatibility, catheter-associated urinary tract infection, and vascular catheter-associated infection. As described elsewhere, perioperative complications were defined as any postoperative adverse event, with or without further surgical intervention, occurring within 30 days of the initial surgery (Schneider et al. 2021).

Exclusion criteria encompassed patients classified as non-operable and those for whom we lacked complete data or surgery-related information. In addition, patients with no brain metastases were removed from the retrospective data analysis. Some patients had a primary suspected diagnosis of metastasis, but a different pathology was found after resection; such patients were not included in the data collection.

The study adhered to the ethical principles outlined in the 1964 Helsinki Declaration and received approval from the Ethics Committee of the University Hospital Bonn (reference no. 067/21). Given the retrospective nature of the study, the acquisition of informed consent from participants was not necessary.

### Radiological evaluation

Perioperative imaging data were obtained and analyzed by an independent neuroradiologist in accordance with our institution’s standards.

### Statistical analysis

The analyses of Data were performed using SPSS-Program (version 25, IBM Corp., Armonk, NY) and PRISM computer software packages. Categorical variables/Parameter were analyzed in contingency tables using Fisher’s exact test. The Mann–Whitney U test was chosen to compare continuous variables as the data were mostly not normally distributed, while non-parametric data are summarized by median values (first quartile—third quartile). Results with *p* < 0.05 were considered statistically significant. *Univariate analysis* (including following factors: primary tumor, median age, sex, location BM, median CCI, perioperative KPS, BMI, ASA Score, Anesthesia art, Blood Loss, anestehsia time, median duration of surgery, early postoperative complications, anticoagulation) *was conducted using Fisher*’*s exact test (two-sided) and the independent t-test. p values* < *0.05 were considered statistically significant*. In addition, in order to determine independent predictors of complications after early or delayed extubation in patients with surgically-treated BM, a backward stepwise method was used to construct a multivariate analysis using a multiple logistic regression model, again with *p* < 0.05 being considered statistically significant.

### Postoperative procedure

Patients were considered for EE based on stable intraoperative hemodynamics, absence of neurological deficits on initial assessment, and satisfactory emergence from anesthesia in the recovery room. Patients exhibiting delayed awakening or respiratory instability, or requiring additional monitoring, were transferred to the ICU for DE. These decisions were made at the discretion of the attending neurosurgeon and anesthesiologist. Postoperative complications, in particular re-intubations, infections or other adverse events, were documented in both groups.

## Results

### Patient characteristics and demographic data

A total of 190 patients were surgically treated for BM and included in the analysis, with 65 (34.2%) in the EE group and 125 (65.8%) in the DE group. The median age was similar between groups (*p* = 0.340), as was sex distribution (*p* = 1.000). Body mass index (BMI) distribution showed a trend towards higher measures (≥ 30) in the DE group (94.4%), although this was not statistically significant (*p* = 0.175). For further details of patients’ characteristics, see Table 1.

**Table 1:** Baseline data comparing patients of both groups, univariate analysis

Total (n = 190)	EE group	DE group	*p* value
No. of patients	65 (34.2%)	125 (65.8%)	
Age (yrs.), median (q1-q3)	65 (54.5–71.5)	63 (56–69)	0.340
Sex			1.000
Female	34 (52.3%)	66 (52.8%)	
Male	31 (47.7%)	59 (47.2%)	
BMI (kg/m^2^)			0.175
< 30	64 (98.5%)	118 (94.4%)	
≥ 30	1 (1.5%)	7 (5.6%)	
Length of stay in days, median (q1–q3)	11 (8–16)	13 (9–20)	0.342
Op duration in min., median (q1–q3)	196 (138–256)	217 (160–269)	0.175
Anesthesia time in min., median (q1–q3)	301 (248–369)	317 (271–360)	0.151
Blood loss in ml, median (q1–q3)	100 (100–150)	100 (100–200)	0.133
Intra-operative transfusion	1 (1.6%)	13 (10.4%)	**0.037**
ASA score			0.437
1 or 2	25 (38.5%)	59 (47.2%)	
3 or 4	40 (61.5%)	66 (52.8%)	
Anesthesia art TIVA	65 (100%)	125 (100%)	
CCI—total			0.134
< 7	26 (40%)	64 (51.2%)	
≥ 7	39 (60%)	61 (48.8%)	
KPS			0.062
< 70%	5 (7.7%)	21 (16.8%)	
≥ 70%	60 (92.3%)	104 (83.2%)	
Anticoagulation	16 (24.6%)	29 (23.2%)	0.481
BM volume in cm^3^, median (q1–q3)	15.5 (9.6–36.4)	18.5 (7–32.5)	0.751
BM location			0.530
Supratentorial	45 (69.2%)	85 (68.5%)	
Infratentorial	20 (30.8%)	39 (31.5%)	
Postoperative primary ventilation (duration) in min., median (q1–q3)	36 (28–55)	160 (95–300)	**0.002**
Pathology			0.190
Lung cancer	25 (38.5%)	60 (48.0%)	
Breast cancer	11 (16.9%)	12 (9.6%)	
Gastrointestinal cancer	12 (18.5%)	14 (11.2%)	
Others	17 (26.2%)	39 (31.2%)	

ASA, American Society of Anesthesiologists; BM, Brain metastasis; BMI, Body mass index; CCI, Charlson Comorbidity Index; COPD, Chronic obstructive pulmonary disease; DE, Delayed extubation; DVT, Deep vein thrombosis; EE, Early extubation; KPS, Karnofsky Performance Scale; Op, Operation; PE, Pulmonary embolism; TIVA, Total intravenous anesthesia

Patients, who were extubated in the recovery room according to our new protocol, have an average ventilation time of 36 min. In contrast, patients who were extubated in the intensive care unit took longer to be extubated (*p* = 0.002).

### Patient-related and disease-related factors associated with early vs delayed extubation

Relevant operation and anesthesia duration were marginally longer for both surgery (217 min) and anesthesia time (317 min) in the DE group, although these were not statistically significant. Blood loss and transfusion were significant in the DE group (*p* = 0.037 for transfusion). Correspondingly, intra-operative transfusion was more frequent in the DE group than in the EE group (10.4% vs 1.6%).

A higher CCI score (> 7) was observed in a larger proportion of patients in the EE group, without significant intergroup differences (*p* = 0.134). The KPS in both groups had similar distributions (*p* = 0.062). The distribution across pathologies (e.g. breast cancer, gastrointestinal cancer) was comparable, with no significant differences between the two groups.

### Postoperative complications and ICU observation/admission

Both groups had similar ICU admission rates and average stay durations, with no significant variations. Relevant postoperative complications were higher in the DE group (9.2% vs 6.1% in the EE group). The postoperative complications are listed in Table 2. Adverse events, such as pneumonia and sepsis, occurred more frequently in the DE group, although without reaching statistical significance. Two patients in the EE group and four in the DE group had to be re-intubated (*p*-value = 0.571).

**Table 2:** Postoperative complications

Postoperative complication	EE group	DE group	p-value
			0.571
Surgery-related			
Hematoma	1	3	
Wound infection	1	0	
Adverse event			
Pneumonia	0	2	
PE/DVT	0	2	
Sepsis	0	1	
Re-intubation	2	4	
Total	4 (6.1%)	12 (9.2%)	

DE, Delayed extubation; DVT, Deep vein thrombosis; EE, Early extubation. PE, Pulmonary embolism

We had one patient in the DE group who had seizures postoperatively due to the pareital location of the metastasis, but this patient had become symptomatic preoperatively due to seizures.

### Multivariable analysis for predictors infkuencing postoperative extubation

**Fig. 1:**
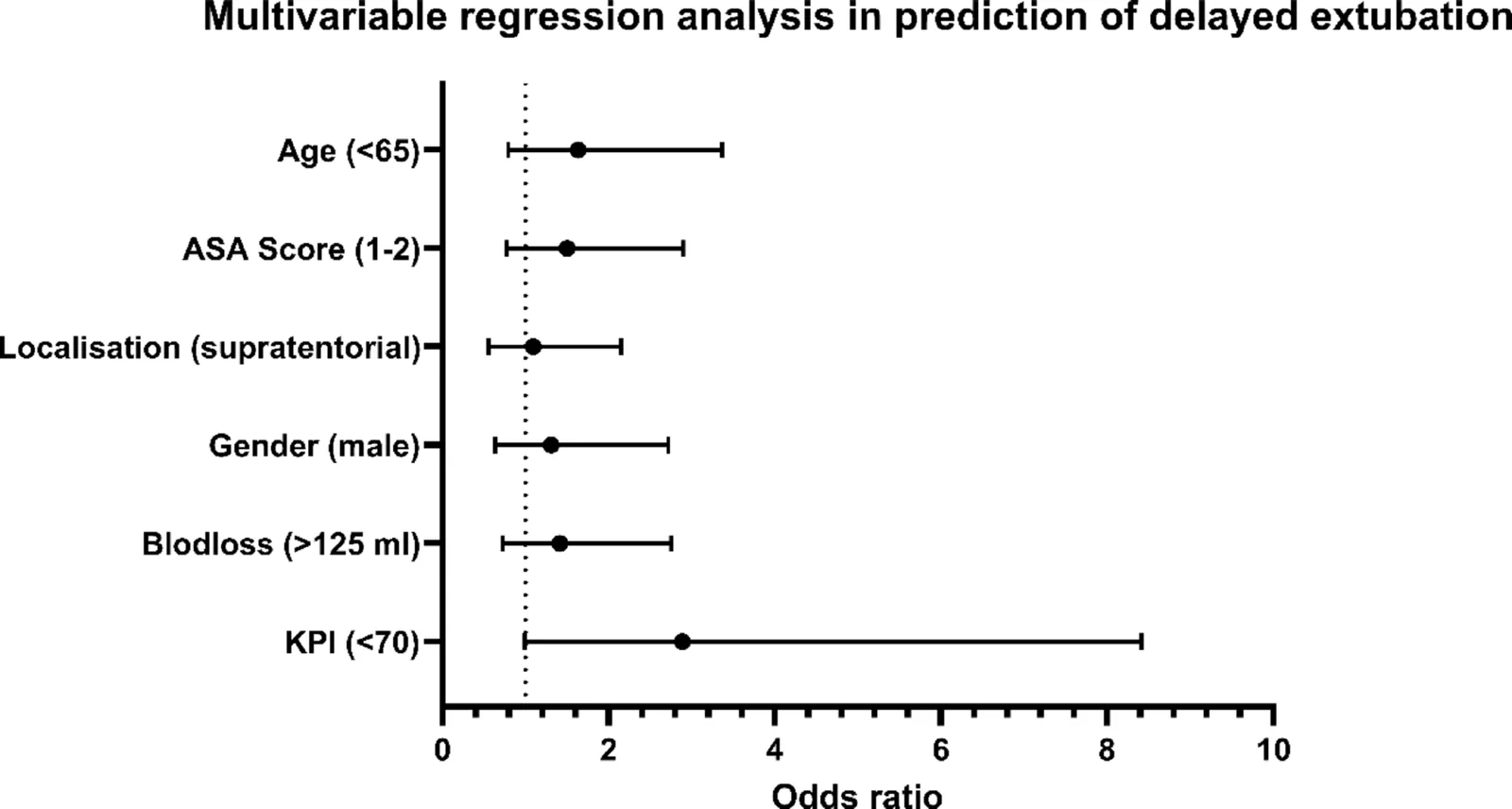
Radar plot depicting patient- and disease-related characteristics dependent on early or delayed extubation. Multivariable logistic regression model displaying odds ratios (OR) with 95% confidence intervals (CI) for various potential predictors of the outcome

We performed a multivariable regression analysis including the variables age, sex, ASA score, Location of tumor, and preoperative KPS, in order to identify independent predictors of early vs delayed postoperative extubation; Fig. 1. No significant factors were identified associated with better outcome in both groups. The aspect of blood loss is in the multivariate analysis without significance.

## Discussion

Extubation and postoperative monitoring of patients in the ICU after major elective surgical treatment of the brain is widespread and standard clinical practice (Ohbe et al. 2022). In our center we deviate from this standard by extubating patients in the recovery room after cranial epilepsy surgery and transferring them to the intermediate care station (IMC) Unless clinically contraindicated, we used the same approach for patients who had undergone surgery for brain metastases.

The aim of this current study is to find out whether earlier postoperative extubation in the recovery room with monitoring of patients in either intensive care or intermediate care posed a risk, or whether this approach was a safe and effective measure.

Postoperative admission to the ICU is routinely used in the surgical world to minimize the risk of postoperative surgical, internal complications and death in patients undergoing major surgery (such as cardiovascular surgery), as well as in high-risk patients undergoing less major surgery (Pearse et al. 2011; International Surgical Outcomes Study g 2016). Utilization of the ICU can reduce mortality in surgical patients through intensive monitoring, experienced staff able to respond quickly, and speedy interventions for abnormalities (Jhanji et al. 2008). Theoretically, patients undergoing elective intracranial surgery who receive delayed extubation and require prolonged mechanical ventilation may have an increased incidence of postoperative pulmonary complications, tracheotomy, re-operation, and prolonged ICU stay, which are associated with mortality (Vidotto et al. 2011). In our cohort, we found a tendency for patients in the early extubation group to have fewer complications than those who were extubated later, but this was not significant.

In our series, we found no influence of tumor entity on the decision of whether patients should be extubated early in the recovery room or later in the ICU, nor did the location of the tumor, whether supra- or infratentorial, have any influence. However, other studies favor monitoring in the ICU, depending on the location and type of tumor (Gupta et al. 2021). Better outcomes, such fewer adverse events, shorter hospital stays and lower hospital costs, are potentially associated with early extubation after elective surgery (Gaudet et al. 2024).

It is well known that body mass index (BMI) is a relevant factor in many surgical studies and has a significant influence on outcomes. We had no overweight patients who had a positive or negative influence on the result, i.e. the 8 obese patients in our series were also treated postoperatively according to the same procedure.. However, several studies show that patients with elevated BMI are at risk of prolonged ventilation, re-intubation or re-operation for infection after elective craniotomy (Dasenbrock et al. 2017a, 2017b).

A retrospective study from Cleveland Hospital showed that, after infratentorial craniotomy, patients who could not be extubated early had higher ASA scores, longer operation times, greater blood loss and longer hospital stays. Furthermore, early extubation in the recovery room after infratentorial craniotomy is feasible. This finding is consistent with our data on early extubation (Cai et al. 2013, 2016; Cata et al. 2011).

Some studies even state that routine admission for elective craniotomy is not necessary for all patients, so that substantial hospital resources can be saved (Hanak et al. 2014).

Intraoperative blood loss was the only significant factor for prolonged ventilation and ICU admission in our univariate analysis. Similar results are found in other studies from other surgical disciplines (Demirci et al. 2017; Lonjaret et al. 2017).

Anesthesia has a potential major impact and a crucial role in postoperative management after resection of a BM. Factors such as appropriate analgesia, early cessation of opioid administration, and balanced perioperative inhalational/intravenous anesthesia have all contributed to improvement in cognitive function and early extubation after craniotomy without raising intracranial pressure (Xu and Vagnerova 2021; Ayrian et al. 2015).

Postoperative complications after neurosurgical procedures can vary; the range of such complications is very wide, and include surgery-related and adverse events. Various possible factors influence complications, such as postoperative bleeding, pneumocephalus after craniotomy, seizures, postoperative delirium, infections, and the age of the patient. The rate of complications reported in the literature is between 2 and 20% (Lynam et al. 2007; Puri et al. 2020; Lee et al. 2024; Bervitskiy et al. 2021; Lenga et al. 2025). This does not differ significantly from our data. As the patients in the DE group do not show any relevant significance in terms of complications, this option is still available as a concept for the medical team who do not have the resource of a recovery room.

## Limitations

The present study has several limitations. Firstly, data acquisition was retrospective; data are therefore subject to well-known and well-described types of bias. Our patients were not blended randomized and their treatment was decided on by the neurosurgeons and anesthesiologists. The number of patients is small, which means the univariate and multivariate analyses may be subject to error. This may lead to further investigations structured to avoid the potential selection bias due to the limited group size in our study. In addition to the limitations mentioned, it should be added that we had fewer comorbidities in the EE group, this weakness may lead us to believe that the result and conclusion are influenced by this, more patients are certainly needed here. Finaly, we did not calculate an effect size based on the retrospective analysis, this weakness may lead us to believe that the result and conclusion are influenced by this, more patients are certainly needed here.

## Conclusions

Our current results demonstrate that there was no significant difference between early and delayed extubation after elective surgical treatment of brain metastasis. There was no disadvantage to extubating patients early in the recovery room with postoperative monitoring in the ICU or IMC. Delayed extubation was primarily dependent in our series on intraoperative blood loss. This measure is naturally associated with postoperative monitoring in the ICU. In summary, early extubation was a safe measure and potentially better organizational performance.

## Data Availability

No datasets were generated or analysed during the current study.

## Abbreviations

AE: Adverse events
ASA: American Society of Anesthesiologists
BM: Brain metastasis
BMI: Body mass index
CCI: Charlson Comorbidity Index
CI: Confidence interval
DE: Delayed extubation
EE: Early extubation
HAC: Hospital acquired condition
ICU: Intensive care unit
IMC: Intermediate care station
KPS: Karnofsky Performance Scale
OR: Odds ratio
PE: Pulmonary embolism
PSI: Patient safety indicator
SD: Standard deviation
